# Supplementary material for: Observational cohort study of the effect of a single lubricant exposure during transvaginal ultrasound on cell-shedding from the vaginal epithelium
Source: PLoS One. 2021 May 3;16(5):e0250153. doi: 10.1371/journal.pone.0250153 (PMC8092793; doi:10.1371/journal.pone.0250153)
Supplement: S1 Table — (DOCX) [file pone.0250153.s001.docx]

**S1 Table. Wilcoxon signed-rank test to evaluate changes in maturity index and cell count from pre-TVUS to post-TVUS, W1-post-TVUS, and W2-post-TVUS among reproductive age women, stratified by pre-TVUS maturity index.**

|  | Pre-TVUS MI | Change from pre-TVUS to post-TUVS | *p*-value | Change from pre-TVUS to W1-post-TUVS | *p*-value | Change from pre-TVUS to W2-post-TUVS | *p*-value |
| --- | --- | --- | --- | --- | --- | --- | --- |
| Maturity Index (MI) | All | -1.01  [-2.15 – -0.24] | **0.0013** | -1.21  [-2.35 – +0.34] | **0.0045** | -0.56  [-1.95 – +0.44] | **0.0273** |
|  | MI > 3 | -1.51  [-2.99 – -0.42] | **0.0002** | -1.79  [-2.78 – -0.45] | **0.0008** | -0.72  [-2.21 – +0.43] | **0.0116** |
|  | MI < 3 | -0.46  [-1.01 – +0.70] | 0.6465 | 0.62  [-0.01 – +0.90] | 0.3863 | 0.25  [-1.34 – +0.82] | 0.9594 |
| Cell Count/10 μL | All | -6,693.1  [-19,767.7 – +0.2] | **0.0022** | -3,781.2  [-12,608.1 – +2,796.8] | **0.0660** | 550  [-7,989.2 – +7,737.0] | 0.8233 |
|  | MI > 3 | -16,452.4  [-21,031.5 – -4,997.3] | **0.0002** | -6,444.5  [-14,439.4 – +1,267.6] | **0.0133** | -128.4  [-13,180.1 – +7,737.0] | 0.7151 |
|  | MI < 3 | -14.3  [-85.0 – +966.4] | 0.8785 | 1,248.9  [-4,811.8 – +8,607.5] | 0.4446 | 642.8  [-40.0 – +14,031.3] | 0.2411 |
