# Supplementary figures and images for: Observational cohort study of the effect of a single lubricant exposure during transvaginal ultrasound on cell-shedding from the vaginal epithelium
Source: PLoS One. 2021 May 3;16(5):e0250153. doi: 10.1371/journal.pone.0250153 (PMC8092793; doi:10.1371/journal.pone.0250153)

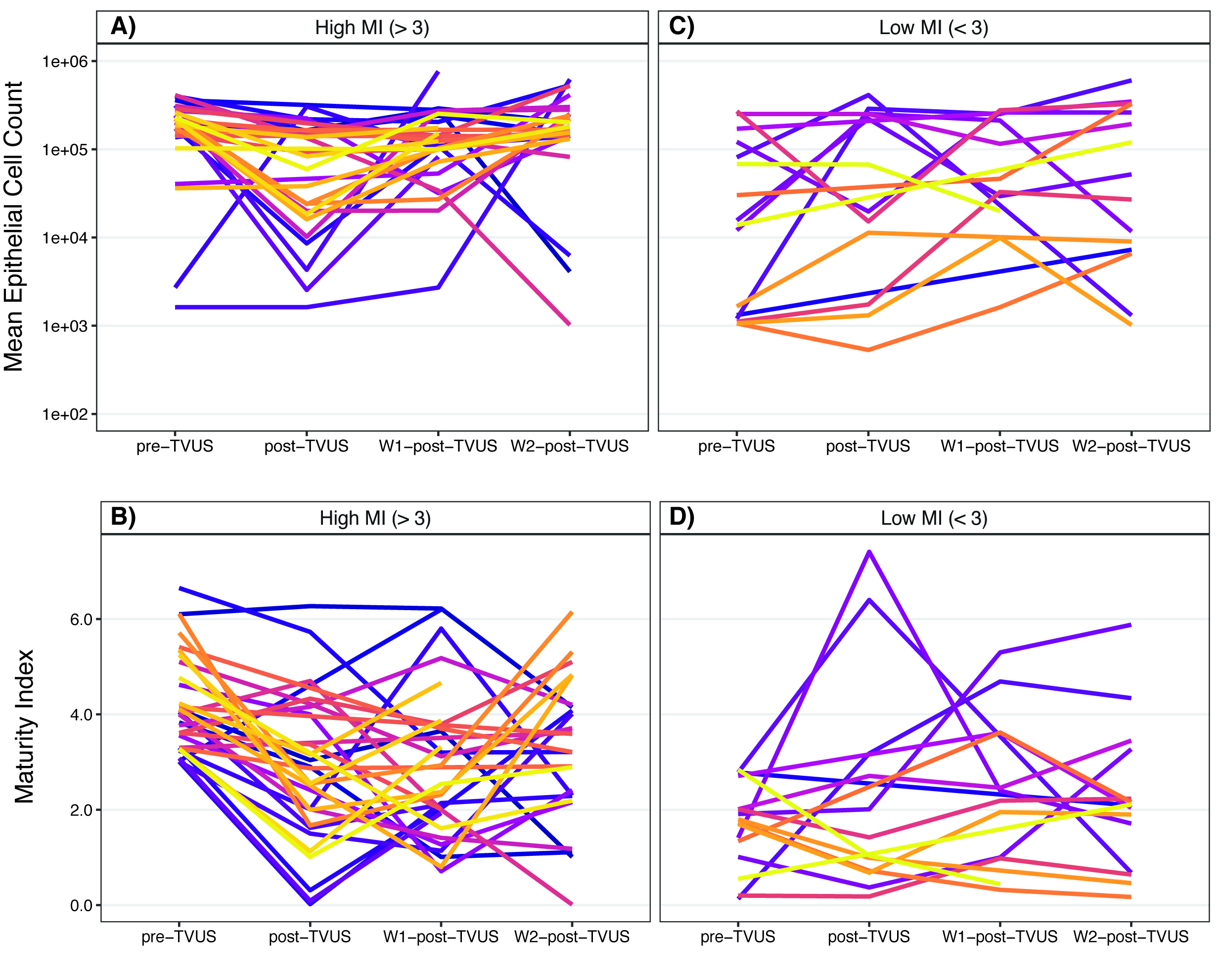

Supplement: S1 Fig — A and B. Plots of individual trajectories for cell-counts and MI, respectively, in women with a pre-TVUS maturity index > 3. C and D. Plots of individual trajectories for MI and cell counts, respectively, in women with a pre-TVUS maturity index < 3. (TIF) [file pone.0250153.s002.tif]
