# Supplementary material for: Observational cohort study of the effect of a single lubricant exposure during transvaginal ultrasound on cell-shedding from the vaginal epithelium
Source: PLoS One. 2021 May 3;16(5):e0250153. doi: 10.1371/journal.pone.0250153 (PMC8092793; doi:10.1371/journal.pone.0250153)
Supplement: Attachment — Submitted filename: Response to Reviewers.docx [file pone.0250153.s003.docx]

We thank the reviewers for their helpful comments and suggestions. Our revisions have led to significant improvements in this manuscript. Below we have listed reviewers’ comments with our responses following in **bold**.

Reviewer 1:

1. The opening premise is that “the other layers of the vaginal epithelium form a physical barrier of dead and dying cells that protect the inner maturing cell layers.” It is unclear what this premise is based on as there are no references provided to this specific point. This actually seems to be a hypothesis for the function of the outer, dead layers of the vaginal epithelium. Other hypotheses also exist, such as that these layers provide a food source for commensal bacteria and/or that they don’t have a specific function and rather simply are a byproduct of the epithelial cell maturation and sloughing process.

**We have removed the above sentence as we did not mean to suggest a singular function for the outer layers of the vaginal epithelium. We have added citations to support the hypothesis that the outer layer provides a food source for *Lactobacillus*, as well as evidence to support an increased risk of infection following detachment and shedding of vaginal epithelial cells.**

2. The authors hypothesize that a single vaginal exposure to commonly used lubricating jelly “accelerates” shedding of the vaginal epithelium. However, in order to test “acceleration” then there must be a comparison to a non-exposed group. The study was not designed this way, rather the women all had multiple variables that may have impacted the measured outcomes (epithelial cell number and maturation index): 1. softcup application #1, 2. TVUS probe, 3. Lubricant.

**Due to NIH funding constraints, we sought to study lubricant use in the context of an observational study and were unable to conduct a clinical trial. The manuscript details an observational study of women presenting for TVUS as it is a controlled environment in which lubricant is used in the conduct of the clinical exam. The exam presented the opportunity to observationally study the effect on the vaginal epithelium of a controlled amount of lubricant (1 single 3g packet) and over a documented time encompassing before and after product used. We asked participants to not use a lubricant at other times during the study and if they did, we instructed them to mark it on their diary. There were only 3 study days in which a participant included in this analysis reported lubricant on their daily diary forms, however just one day which coincided with soft cup collection. Herein, we describe the pre- to post-observational changes in cell shedding following a single use of lubricant during TVUS.**

**1. Softcup: The softcup was used during all sampling time points and would not impact change in either MI or cell count from the pre-TVUS timepoint to the three post-TVUS time points. In addition, women who experienced a decrease in maturity index or cell count following TVUS had those metrics recover at later timepoints, suggesting that the softcup device is not responsible for the changes observed.**

2. TVUS probe: It would not be possible to determine the effect of a TVUS probe alone on maturity index or cell count as a TVUS procedure will always include the use of a lubricant in conjunction with the wand. In this sense, the exposure of lubricant + TVUS wand reflects typical use in clinical practice.

3. Lubricant: We have clarified in the text that lubricant exposure was incidental to TVUS.

3. Further, the vaginal epithelium is clearly hormonally responsive (drastic changes from pre-pubertal-->reproductive age-->menopausal and there are more subtle though still quite perceptible changes in reproductive aged-women through the menstrual cycle as well as with initiation and use/changes in exogenous hormone exposure (hormonal contraceptives)), yet there was no characterization of participants based on age/menopausal status, phase of menses, type of contraception use, etc—all of which are critically important to understand.

The authors state that a strength of the study is the longitudinal within-woman analysis—however the authors do not account for the fact that naturally cycling women and women on cyclic hormonal contraceptives are not in a static state over time with respect to the outcomes of interest—thus, the purported strength is actually a weakness for evaluation of these outcomes. This could have been addressed with a study design that considered hormonal status and timing of collections, however, this study design did not account for these confounding changes.

It would actually be quite interesting to have conducted a study evaluating the epithelial cell numbers and maturity index in cohorts of women separated out by hormonal status: pre-menopausal, naturally cycling in follicular phase; pre-menopausal, naturally cycling in luteal phase; pre-menopausal, on progestogen-only hormonal contraception; pre-menopausal, on combined progestogen/estrogen containing hormonal contraception; post-menopausal. This information could then inform understanding a major factor driving the maturity index, which the authors then used to ‘bin’ the participants. Indeed, the results demonstrate that the only women with significant changes after the ultrasound/lubricant exposure, were those with high MI pre-ultrasound. These women are likely to represent a more hormonally similar grouping, which would be important to know.

**We agree with the reviewer’s assessment, however, a study that stratifies on baseline entry criteria would require a substantial sample size. Unfortunately, we are limited to the 50 women reported in this manuscript. We have emphasized the limits of analysis due to sample size in the discussion and have indicated that our data are preliminary and require future studies.**

4. Thus, the study consisted of an uncharacterized, likely quite heterogeneous in terms of hormonal status, women who all had 3 exposures, and any observed results may have been attributed to any of these exposures, yet the authors conclude that the observed results are based only on the lubricant exposure. This is very problematic.

**Use of hormonal contraception was not associated with pre-TVUS MI, and neither was self-reported vaginal bleeding on the day of TVUS (Table 1). There was very little within-woman variability in self-reported vaginal bleeding over the four sampling timepoints, probably due to the short timeframe in which samples were collected. We have clarified that the average number of days between the pre-TVUS timepoint and the W1-post-TVUS and W2-post-TVUS timepoints were 4 and 7 days, respectively, and 90% of women included in this analysis collected all four samples within a 10-day window. We did find that menopausal status (pre-menopausal versus peri/post-menopausal) was associated with pre-TVUS MI (Table 1). We have noted this finding in the results and in the discussion.**

**We repeated the analysis after excluding peri/postmenopausal women in order to examine the association between lubricant use and MI and cell count in a more hormonally-similar group. The results were largely unchanged, with significant decreases in MI and cell count among reproductive-age participants with a high pre-TVUS MI, and no changes among reproductive-age participants with a low pre-TVUS MI. We have added these results to the manuscript text and as Supplemental Table 1.**

5. Omission of evaluation of the vaginal microbiota, another significant factor contributing to the vaginal microenvironment. In the discussion, the authors do allude to the microbiota having potential impacts on the MI, which is true—indeed it would be important to understand the contributions to the MI from the hormonal state and microbiological state independently.

**This study aims uses preliminary data to address whether there is an association between lubricant use and changes in maturity index or cell shedding. It is certainly possible that this association is mediated by the composition of the vaginal microbiota if lubricant has an effect on the microbiota. Future studies with a larger sample size should explore this hypothesis.**

6. The statement that a single exposure to vaginal lubricant ‘may increase infection susceptibility’ is overreaching and inappropriate given that the study did not assess this in any way.

**We have changed the wording in our conclusion to suggest that future studies should explore whether changes in vaginal epithelial barrier integrity may increase inflammatory status and susceptibility to reproductive tract infections, and to clarify that this outcome was not assessed in our analysis.**

Reviewer 2:

1. Clarify if exclusion criteria and STI results were self-report, or clinician/lab confirmed.

**Decision to exclude was based on laboratory-confirmed STI results, and this is now clarified in methods.**

2. It is stated that the study continued for 9 weeks, but the paragraph on sample collection and the results only show data for 4 time points (pre, 6-12 h, 1 week, 2 weeks). Was data from post 9 weeks part of the study, and if so clarify where?

**These four time points were chosen after initial experiments using all samples demonstrated that the effect of lubricant was limited to the earliest time points.**

3. What did participants do to carry their -20C samples from home to the clinic to keep them frozen?

**We have updated the methods to clarify that participants stored their samples in their home freezers, and were given coolers with ice packs to transport samples from their home freezers to study appointments.**

4. Cell counts: These were performed on cells thawed from the Softdisc collections. But no DMSO or other cell membrane preservative appears to have been included in the collections/freezing. Could the authors explain why cell counts could be performed using the methods they describe as usually freezing of cells lyses membranes and reduces/alters cell counts? Or perhaps epithelial cells freeze well and can be counted following freezing in absence of DMSO? Similarly for maturity index, is this something that is stable to freeze thaw? If it’s based on cytoplasm/nuclear ratio, if cell membranes rupture during freeze/thaw, is cytoplasm volume altered?

**Control experiments showed that freezing undiluted vaginal fluid samples does not cause significant changes in cell counts or maturity index scores compared to the fresh condition. Serum albumin is a widely used cryoprotectant, and the relatively high protein content of vaginal fluid presumably functions in a similar fashion.**

5. The text of Fig 2 and 3 legends is a bit confusing. Although the Y axis labels and title of the Fig legend indicate the graphs refer to “change” the text reports “cell count” or “maturity index” not “change in cell count” or “change in maturity index”, so I spent a while looking in the Figs for the actual #s.

**The figure legend for Figure 2 has been changed to clarify that changes from pre-TVUS cell count or cell maturity index are reported. Figure 3 does not refer to a change in cell count or cell maturity index, and the axis titles and figure legend reflect this.**

6. The authors could add (end of para 2) that future studies with earlier time points (within a few hours) would confirm the theoretical increased VE shedding that the authors think they missed.

**We have added this suggestion for future research in the discussion.**
