# Supplementary material for: Observational cohort study of the effect of a single lubricant exposure during transvaginal ultrasound on cell-shedding from the vaginal epithelium
Source: PLoS One. 2021 May 3;16(5):e0250153. doi: 10.1371/journal.pone.0250153 (PMC8092793; doi:10.1371/journal.pone.0250153)
Supplement: Attachment — Submitted filename: Response to Reviewers.docx [file pone.0250153.s004.docx]

**Reviewer 1:**

1. I would request a reframing of the title because although there was only a single exposure to gel, the gel was not the only exposure for the vagina. Within the body of the text, the authors did adjust the language of the TVUS probe slightly, to now read “...the effect of a single lubricant exposure incidental to TVUS on cell-shedding from the VE...” This needs to be made even more explicit. I recommend reframing to include the probe in the exposure as follows: “...the effect on cell- shedding from the VE of a single exposure to lubricant gel administered on a TVUS probe ... This is truly the exposure. To address the issue with not having a control to assess the impact of the softcup ‘scraping/squeegee’ effect on the vaginal epithelium (for instance, best would be to have had a parallel cohort of women who performed all of the softcup collections at the same times and did not have the gel/TVUS exposure), this needs to be clearly stated in the discussion as a weakness of this study and an acknowledgement that removal of VE cells by softcup squeegee could have contributed to and/or caused the reduction in epithelial cell number and/or MI demonstrated by the data. Indeed, as noted by the authors, this collection method recovers “large volumes of CVF.” The data are what the data are—they simply need to be reported in a non-biased manner with all possibilities for the observed findings considered and discussed.

**We have re-titled the manuscript “OBSERVATIONAL COHORT STUDY OF THE EFFECT OF A SINGLE LUBRICANT EXPOSURE DURING TRANSVAGINAL ULTRASOUND ON CELL-SHEDDING FROM THE VAGINAL EPITHELIUM”. This revised title emphasizes that the lubricant exposure occurred in the context of a transvaginal ultrasound exam.**

**We have also made the other suggested edits to the introduction and discussion. Specifically, we have emphasized that this observational study cannot isolate the effects of the lubricant from other possible effects of the probe or SoftDisc collection device, and that the higher proportion of unusable samples at the post-TVUS timepoint (compared to the other timepoints) may be explained by its proximity to the pre-TVUS timepoint. The text also notes that *in vitro* studies with lubricant as the only exposure provide further support that lubricant are the likely source of the toxic effects on the vaginal epithelium.**

2. In the abstract, rather than stating, “We determined whether a typical clinical lubricant accelerates shedding of mature epithelial cells, exposing immature cells.”, consider reframing as “We hypothesized that exposure to a typical vaginal lubricant, used for clinical pelvic exams and during transvaginal ultrasonography (TVUS), may contribute to shedding of mature epithelial cells, exposing immature cells. To test this hypothesis, we enrolled women scheduled to undergo TVUS examination for a variety of indications and who were willing to undergo sampling of cervicovaginal fluid immediately before and at three timepoints after the single exposure to lubricant and TVUS (6-12 hours, within one week, and two weeks after).” Please make careful note that the word ‘accelerates’ is problematic as that implies a comparator, and in this study there are none. Same issue in the first sentence of the Discussion, which describes both ‘accelerated’ and ‘increased’ as findings to measures without comparators.

**We have made these suggested edits. See the abstract and below excerpt from the discussion**

**Discussion, first paragraph: *“We hypothesized that a single vaginal lubricant exposure, as part of a TVUS protocol, would promote shedding of VE cells...”***

3. Please add references for the statement, “The balance between detachment/shedding and proliferation/maturation processes may influence susceptibility or resistance to reproductive tract infections.”

**We have reworded this sentence. It is hypothesized that disruption to this balance may increase susceptibility to reproductive tract infections and we have provided citations for this hypothesis.**

**Introduction, first paragraph: *“Detachment/shedding of the outer layer of mature cells in excess of proliferation/maturation rates will expose the underlying living cells [Figure 1C], and it has been hypothesized that this may influence susceptibility or resistence to reproductive tract infections (5, 6).”***

4. Figure 1. A—Please re-word “as they move up” and “through the parabasal...” as these phrases are non-scientific, vague, and confusing. Figure 1. B—Please do not start with “These” as it is unclear to what this refers. Be specific in your written language.

**We have re-worded the legend for Figure 1 as pasted below:**

**Figure 1.**

**A. The vaginal epithelium undergoes morphological changes during maturation. As cells mature, they migrate from the basal layer to the parabasal and intermediate layers to the superficial layer from which they are shed.**

**B. Morphological changes of the vaginal epithelium during maturation include decrease in nuclear and increase in cytoplasmic sizes, which provides the basis of the Maturity Index value (values shown are representative of each layer).**

**C. Shedding of immature cells indicates depletion of superficial cells, and loss of structural integrity of the vaginal epithelial barrier.**

5. Participants—please add that the exclusion criteria “lubricant use in the week prior to TVUS” was by self-report. Please confirm that at the enrollment visit there was nothing placed in the vagina prior to the softcup collection of vaginal fluid.

**We have clarified the lubricant use is self-report. We do not utilize samples from the enrollment clinical visit, but we do use one sample from the post-TVUS follow-up visit. Nothing is placed in the vagina prior to the softcup collection – the clinical exam, which utilizes a speculum lubricated with water, occurs following sample collection.**

**Methods (participants): “...*and self-reported lubricant use in the week prior to TVUS. Participants self-collected vaginal samples during the week prior to TVUS and for nine weeks following TVUS, and attended three clinical visits at enrollment, within one week of TVUS, and at the completion of the study. No lubricant was used during the three clinical visits, and sample collection occurred prior to the pelvic exam.”***

6. Was this study registered on ClinicalTrials.gov? If so, please add the registration number to the manuscript.

**This study was not registered on ClinicalTrials.gov. It was an observational study, not a clinical trial.**

7. Do you have information about the length of time between the TVUS and the collection of CVF ‘at bedtime on the day of ultrasound’? What was the mean and median number of hours between these two timepoints?

**Participants did not record sample collection time at home. Participants were instructed to take the post-TVUS samples the evening of the TVUS appointment. All TVUS appointments occurred between 7:00 am and 4:00 pm, and so 6-12 hours is a reasonable estimate of the time between the TVUS appointment and the post-TVUS sample.**

**Methods (Sample collection and processing): *“Participants’ cervicovaginal fluid (CVF) was sampled (1) immediately before TVUS (pre-TVUS), (2) at bedtime (approximately 6-12 hours after TVUS) on the day of the ultrasound, (post-TVUS), (3) at a follow-up visit within one week of TVUS (W1-post-TVUS) and (4) within two weeks of TVUS (W2-post-TVUS).”***

8. Missing 22% of the post-TVUS samples is significant enough to deserve a comment in the weaknesses section of the discussion.

**We have clarified in the methods that 22% of samples were not “missing”. We restricted the analysis to participants who returned all 4 samples and who had a usable volume for the pre-TVUS sample (N= 50 women and 200 samples). In 22% of post-TVUS samples, there was not a usable volume of sample in order to determine cell count and maturity index. We have mentioned this as a limitation in the discussion. Of the 29 samples which did not have a usable volume for experiments, 13 (45%) belonged to women with a low pre-TVUS MI and 16 (55%) belonged to women with a high pre-TVUS MI. The text now reads:**

**Methods (Statistical analyses): “*Not all women provided samples with a useable volume of CVF at all four time points (22% at post-TVUS, 16% at W1-post-TVUS, and 18% at W2-post-TVUS did not have usable volume), therefore n is reported separately for each comparison.”***

**Discussion: 2^nd^ to last paragraph*: “Lastly, we were unable to make comparisons with pre-TVUS MI and cell-count for 22%, 16%, and 18% of samples at the post-TVUS, W1-post-TVUS, and W2-post-TVUS timepoints because the SoftDisc^TM^ did not recover a large enough sample volume for experiments. It is possible that removal of VE cells by the SoftDisc at the pre-TVUS sample may have contributed to the higher proportion of unusable samples at the post-TVUS timepoint. 45% of samples without a usable volume occurred among women with a low pre-TVUS MI, and 55% occurred among women with a high pre-TVUS MI.”***

9. Beware that not all ‘hormonal contraception’ is comparable, particularly as it relates to impacting the genital milieu. Women using depot medroxyprogesterone acetate will have markedly thinned epithelia and endometria. Women using hormonal IUDs will also have markedly thinned endometria. Clearly, this study enrolled women using hormonal IUDs. Perhaps adding the various contraceptive methods to Table 1 under ‘Current use of any hormonal contraception’ would be useful.

**No participants included in this analysis reported current use of DMPA. We have added type of hormonal contraception used in Table 1. Note that a similar proportion of participants in the low MI and high MI groups reported using a hormonal IUD (11% versus 13%). The percentages for type of hormonal contraception further break down the 29% and42% current users.**

**From Table 1:**

|  | MI < 3  N (%) | MI > 3  N (%) | p-value |
| --- | --- | --- | --- |
| Current use of hormonal contraception  *Type of hormonal contraception*  Oral contraceptive  Intrauterine device  Implant | 5 (29%)      2 (11%)  2 (11%)  1 (6%) | 13 (42%)        4 (13%)  4 (13%)  5 (16%) | 0.65^b^ |

10. Under ‘Comparison of women with high and low pre-TVUS Maturity Index at W2-post- TVUS’, paragraph starting ‘Maturity index (MI)...’, the verb ‘was’ should be ‘were’ in order to agree with the pleural ‘MIs’.

**We have made this change.**

**Results (Comparison of women with high and low pre-TVUS Maturity Index at W2-post-TVUS): *“The MIs of W2-post-TVUS samples from women with low pre-TVUS MIs were significantly lower than that from women with high pre-TVUS MIs…”***

11. Discussion, paragraph starting, “The epithelial cell changes...” contains a sentence,
    “These participants began the study with optimal and balanced VE proliferation/maturation and detachment/shedding, the single use of vaginal lubricant incidental to TVUS seems to have caused a significant disruption of that balance.” I don’t see any evidence presented demonstrating “optima and balanced VE proliferation/maturation and detachment/shedding”—what is this based upon? What are the criteria for having an optimal balance? This statement is problematic. Also, the next sentence talks about ‘disruption’, which may or may not be accurate given the absence of data about what is normal at various points in the menstrual cycle. Overall, using language that is purely descriptive, such as “In W1- and W2-post-TVUS samples, we observed persistently low cell numbers and low MIs compared to baseline” and avoiding language that demonstrates bias, such as ‘disruption’, would be preferable. In the discussion it is fair to then hypothesize that the persistently low cell numbers and MI may represent significant and persistent disruption in the epithelium by using language such as this that clearly frames a hypothesis and not stated as fact.

**We have re-written this portion of the discussion. We are unable to determine with a single measurement whether these participants have balanced VE proliferation/maturation and detachment/shedding. We intended to convey that, because it is hypothesized that shedding of mature VE cells is an important mechanism of host defense against infection, the observed shift from shedding mature VE cells prior to TVUS to shedding less mature VE cells afterwards suggests this mechanism may be impaired. The text now reads:**

**Discussion, paragraph 4*: “***

***The epithelial cell changes observed in this study occurred almost entirely in participants whose pre-TVUS sample had high MI (> 3). It has been hypothesized that exfoliation of mature superficial cells is one mechanism by which the vaginal epithelium provides protection against infection (5). However, shedding of immature cells reflects a depletion of superficial cells and a loss of vaginal epithelial barrier integrity. Following the single use of vaginal lubricant during TVUS, we observed the shedding of fewer, and more immature, VE cells in both the W1-post-TVUS and W2-post-TVUS samples, suggesting this proposed protective mechanism may be disrupted.”***

**As for what is normal at various times in the menstrual cycle, Patton *et al* found evidence for small changes in the mean number of vaginal epithelial cell layers throughout the menstrual cycle (27.8 on days 1-5, 28.1 on days 7-12, and 26.0 on days 19-24), and it was noted that these differences were unlikely to be clinically significant [Patton *et al, Am J Obstet Gynecol*, 2000]. We have added this finding.**

**Discussion, paragraph 7: *“Patton et al found evidence for only small changes in the mean number of epithelial cell layers throughout the menstrual cycle (27.8 on days 1-5, 28.1 on days 7-12, and 26.0 on days 19-24) (4)”***

12. Please re-word the vague and grammatically incorrect sentence, “This is not to say that these women’s VE is not affected by lubricant, only that these effects cannot be measured by our methodology.”

**We have clarified this statement in the discussion. The sentence now reads:**

**Discussion, paragraph 5: *“In other words, in women whose VE is already depleted of mature cells, lubricant exposure does not appear to produce much discernable change in the amount or maturity of cells shed as measured by our methodology.*”**

13. With respect to the following author responses: “1. Softcup: The softcup was used during all sampling time points and would not impact change in either MI or cell count from the pre- TVUS timepoint to the three post-TVUS time points. In addition, women who experienced a decrease in maturity index or cell count following TVUS had those metrics recover at later timepoints, suggesting that the softcup device is not responsible for the changes observed. Response to Reviewers 2. TVUS probe: It would not be possible to determine the effect of a TVUS probe alone on maturity index or cell count as a TVUS procedure will always include the use of a lubricant in conjunction with the wand. In this sense, the exposure of lubricant + TVUS wand reflects typical use in clinical practice.” 1. Softcup—if the softcup itself (which is an exposure) caused the removal of VE cells, prior to the gel/TVUS probe exposure, the change in MI and/or cell count caused by the softcup would be seen as a change from baseline to follow-up. The removed cells would be in the baseline cup—thus making that measurement ‘high’ relative to the follow-up measurement. 2. Agree that the exposure of the TVUS probe cannot be divorced from the gel, which is precisely why together, they need to be described as the ‘exposure’ and not just the gel.

**We now discuss these methodological limitations in the discussion, and have clarified throughout the manuscript that the true exposure is vaginal lubricant use during TVUS (also see responses to Comment 1 and 8).**

**It is true that the pre-TVUS and post-TVUS time points occur closer in time together (within 6-12 hours versus multiple days between), and that this may help explain the higher proportion of unusable samples at the post-TVUS timepoint compared to the later timepoints. Among the high MI group, we did see the decrease in cell count persist through the W1-post-TVUS timepoint and the decrease in MI persist through the W2-post-TVUS timepoints. These samples were not taken in close proximity to the previous sample, and so we believe it is less likely that these decreases are explained by the use of the Softcup at the prior timepoint. Still, we agree with the reviewer that it should be mentioned that we cannot attribute these findings to the use of lubricant alone. We have added the following text to the discussion:**

**Discussion, paragraph 3: *“Additionally, while it is not possible with this observational study design to separate the effects of lubricant from other possible factors, including effects of the TVUS probe or SoftDisc^TM^ collection device, in vitro studies support that hyperosmolal lubricants alone are capable of reducing epithelial cell barrier integrity and causing tissue morphological damage (28, 37).”***

**Reviewer 2:**

1. Only request is in the methods section to indicate that the freeze/thaw method is assumed to not affect the assays, based on what the authors said in their response to my question.

**We have made the following addition to the methods section:**

**Methods (Sample collection and processing): “*Previous control experiments suggested that freezing undiluted vaginal fluid samples did not affect cell count or maturity index scores compared to fresh condition.”***
