# Supplementary material for: Observational cohort study of the effect of a single lubricant exposure during transvaginal ultrasound on cell-shedding from the vaginal epithelium
Source: PLoS One. 2021 May 3;16(5):e0250153. doi: 10.1371/journal.pone.0250153 (PMC8092793; doi:10.1371/journal.pone.0250153)
Supplement: Attachment — Submitted filename: Response to Reviewers.docx [file pone.0250153.s005.docx]

**Abstract:**

1. Please correct the punctuation in the 1st sentence. The first sentence seems disconnected from the rest of the abstract and as currently written the connection of this sentence to the rest of the abstract may not be clear for all readers. This can be resolved by adding the concept that the outer layers are important because..

**The sentence now reads: *The outer layers of the vaginal epithelium (VE) are important because they accumulate glycogen which, under optimal conditions, Lactobacillus spp. consume to grow and acidify the vaginal microenvironment with lactic acid.***

2. Please re-word the sentence that starts, “The clinical lubricant, EZTM lubricating jelly (Chester laboratory),…” As written, this sentence is awkward. You could say that vaginal lubricants used clinically commonly have high osmolality, pH~5.5, and contain bactericides.

**The sentence now reads: *Clinical vaginal lubricants are similar to commercial lubricants, and often have a high osmolality or pH, and contain bactericides such as methylparaben and propylparaben.***

**Introduction:**

1. Change ‘upwards’ in 3rd sentence—this directionality does not make physiologic sense. Perhaps ‘outwards’ or ‘more distal’ could substitute.

**We have changed “upwards” to “outwards”**

2. 2nd sentence of 2nd paragraph: recommend alternate word choice for ‘seem necessary’

**We have changed “seem necessary” to “promote”, and have added an additional reference to support this sentence, which now reads: *Glycogenated superficial cells and free glycogen in vaginal fluid may promote colonization by vaginal Lactobacillus spp. (5, 6), which produce lactic acid and acidify the vagina (pH <4) (7).***

**Methods:**

1. Participants, 1st sentence: change the tense of ‘seeks’ to match the rest of the sentence that is appropriately past tense.

**We have changed “seeks” to “sought”**

2. Previous control experiments (RE: freezing of CVF)—please add ‘(data not shown)’

**We have added “(data not shown)”.**

3. Were all of the oral contraceptive users on estrogen-containing pills? If so, please change to ‘combined oral contraceptives. If not or unknown, keep as oral contraceptives.

**The survey question for contraception had “birth control pill” as an answer choice; we do not know whether participants were on combination or progestin-only birth control pills. We have left it as “oral contraceptives”.**

**Results:**

1. Paragraph 3, 1st sentence: “…indicating depletion of mature…” Please consider changing ‘depletion’ to ‘paucity’ since depletion suggests a change and there is no baseline sampling and thus no comparator. Use of ‘paucity’ also would parallel your appropriate use of ‘predominance’ rather than suggesting that there is an increase.

**We have changed “depletion” to “paucity”.**

2. Please remove the bias from sentence #1 in Comparison of women with high and low pre-TVUS Maturity Index at W2-post-TVUS. Consider: ‘To assess correlation between the initial epithelial state (high or low pre-TVUS MI) and epithelial state at W2 post TVUS.

**We have rewritten this sentence and it now reads: *To assess whether there was any association between the initial condition of the epithelium and the condition of the epithelium two weeks following lubricant exposure, we also compared W2-post-TVUS samples from women with high pre-TVUS MI to samples from women with low pre-TVUS MI.***

3. It looks like women with low or high pre TVUS MI still had low or high W2 post TVUS MI. Did you assess the change in MI pre-post TVUS? That is, assessing change from baseline for each woman rather than the inter-participant evaluations that you are reporting here? Indeed, this is what is essentially shown in Supplementary Fig 1. Any significant changes? Based on just an eyeball look, it does not appear so, if true, this is important.

**Aside from the comparison of W2-post-TVUS MIs and cell-counts in women with a low versus high pre-TVUS MI, all comparisons reported are within-woman pairwise comparisons to the pre-TVUS sample using a Wilcoxon signed rank test. The Methods section states:**

***Cell-count and MI of each pre-TVUS sample were compared, pairwise, to each participant’s cell-count and MI of her corresponding post-TVUS, within one week (W1-post-TVUS) and within two weeks (W2-post-TVUS) samples.***

**Supplementary Figure 1 shows, for each woman, the cell-count or MI at each timepoint, whereas Figure 2 shows the distribution of within-woman changes in either cell-count or MI compared to pre-TVUS for each follow-up visit, as well as which of these changes were significant. For each woman, the cell-count and MI at each timepoint following exposure to lubricant (post-TVUS, W1-post-TVUS, and W2-ost-TVUS) are compared to her own cell-count and MI before exposure to lubricant (pre-TVUS).**

**Discussion:**

1. Second paragraph, sentence 3—please correct grammar. Also, please take great care to not over-state your findings. This study design is imperfect and the findings are not overly convincing, therefore to state as fact, such as “…resulting in depletion of fully mature cells, reduction of cells available for shedding, and increased exposure/loss of immature cells,…” is overstating the findings of this study

**We believe that the significant within-woman decreases in cell-count and MI observed in the post-TVUS samples compared to paired pre-TVUS samples, together with the other research cited, lends some support to the statement that lubricant may induce rapid shedding of VE cells. However, we have re-written this paragraph to emphasize that that is only one possible explanation for what was observed in this study regarding changes in cell count and MI (and the next paragraph further discusses some of the limitations of this observational data). We have also suggested that future studies could continue to “investigate”, rather than “confirm”, whether rapid loss occurs. The paragraph now reads:**

***Our original expectation was that CVF samples taken approximately 12 hours after TVUS would contain more VE cells than samples taken immediately before TVUS, indicating increased detachment/shedding. In fact, participant’s post-TVUS samples contained significantly fewer cells than their pre-TVUS samples, and post-TVUS samples also had a significantly lower MI than paired pre-TVUS samples. One possible explanation for these findings is that rapid detachment/shedding and subsequent clearance of VE cells may have occurred very soon after lubricant exposure, resulting in the observed decreases in cell maturity. This rapid loss hypothesis is supported by published in vivo research showing that rectal epithelial denudation caused by hyperosmolal lubricants increases within 1.5 hours of lubricant application (32). Future studies could include additional sampling time-points immediately following exposure to lubricant to investigate the rapidity of the shedding response.***
